# Reemergence of Vaccinia Virus during Zoonotic Outbreak, Pará State, Brazil

**DOI:** 10.3201/eid1912.130589

**Published:** 2013-12

**Authors:** Felipe L. de Assis, Wagner M. Vinhote, José D. Barbosa, Cairo H.S. de Oliveira, Carlos M.G. de Oliveira, Karinny F. Campos, Natália S. Silva, Giliane de Souza Trindade, Jônatas

**Affiliations:** Universidade Federal de Minas Gerais, Minas Gerais, Brazil (F.L. de Assis, G. de Souza Trindade, J.S. Abrahão, E.G. Kroon);; Universidade Federal do Pará, Pará, Brazil (W.M. Vinhote, J.D. Barbosa, C.H.S. de Oliveira, C.M.G. de Oliveira, K.F. Campos, N.S. Silva)

**Keywords:** vaccinia virus, orthopoxvirus, viruses, zoonoses, reemergence, Amazon Region, Pará State, Brazil

## Abstract

In 2010, vaccinia virus caused an outbreak of bovine vaccinia that affected dairy cattle and rural workers in Pará State, Brazil. Genetic analyses identified the virus as distinct from BeAn58058 vaccinia virus (identified in 1960s) and from smallpox vaccine virus strains. These findings suggest spread of autochthonous group 1 vaccinia virus in this region.

Over the past decade, several exanthematous vaccinia virus (VACV) outbreaks that affected dairy cattle and rural workers have been reported in Brazil. During outbreaks, lesions developed on teats and udders of dairy cattle and caused a decrease in milk production (*1**,**2*). Infected milkers usually had lesions on their hands; the infection was apparently transmitted by unprotected contact with infected cattle (*1**,**2*).

Molecular studies have shown that autochthonous VACVs from Brazil (VACV-BR) can be divided into 2 groups: group 1 and group 2 (*3**,**4*). Group 1 includes isolates Cantagalo, Araçatuba, Passatempo, Guarani P2, Mariana, and Pelotas 2; group 2 includes isolates Guarani P1, Pelotas1, and BeAN58058 (BAV). This molecular dichotomy is also reflected in certain biologic properties of the isolates, including virulence in the BALB/c mouse model and plaque phenotype in BSC-40 cells (*1*). Although each VACV strain has unique genetic characteristics, most of them are similar to each other within the same group, especially those belonging to group 1; they most likely share a common ancestor. Although some researchers believed that VACV vaccine strains could have spread from humans to domestic animals and adapted to the rural environment (*2*), recent studies have suggested an independent origin for VACV isolates from South America, which is distinct from vaccine strains used in South America during the World Health Organization vaccination campaign. (*3**,**4*).

Despite emergence of VACV in the past decade, VACV was also isolated during the 1960s and 1970s during government efforts to investigate emerging viruses in forests in Brazil (*5**–**7*). One of those isolates, BAV, was obtained in 1963 from the blood of a rodent in Pará State in the Amazon region of Brazil that belonged to the genus *Oryzomys* (*6**,**7*). BAV was characterized during the 1990s, and restriction pattern and nucleotide sequence data supported its classification as a VACV (*6*). However, since its isolation, VACV circulation has not been reported in Pará State, even after VACV outbreaks in southeastern Brazil (*1*). 

In this report, we describe reemergence VACV during a severe exanthematous outbreak in Pará State, 47 years after isolation of BAV. Our molecular data showed that this new VACV isolate clusters with group 1 VACV-BR isolates, which is the same VACV clade related to most viruses that caused zoonotic outbreaks in rural areas of Brazil in the past decade.

## The Study

**Figure 1:**
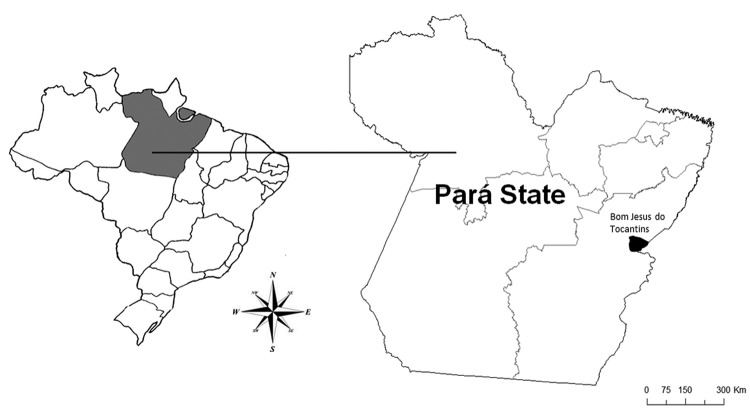
Bom Jesus do Tocantins County (black) in Pará State (gray), Brazil, where an outbreak of bovine vaccinia occurred in 2010.

The outbreak was reported in July 2010 in Bom Jesus do Tocantins County (5°2′ 60″S, 48°36′36″W), Pará State, in the Amazon region of Brazil (Figure 1, Appendix).

**Figure 2:**
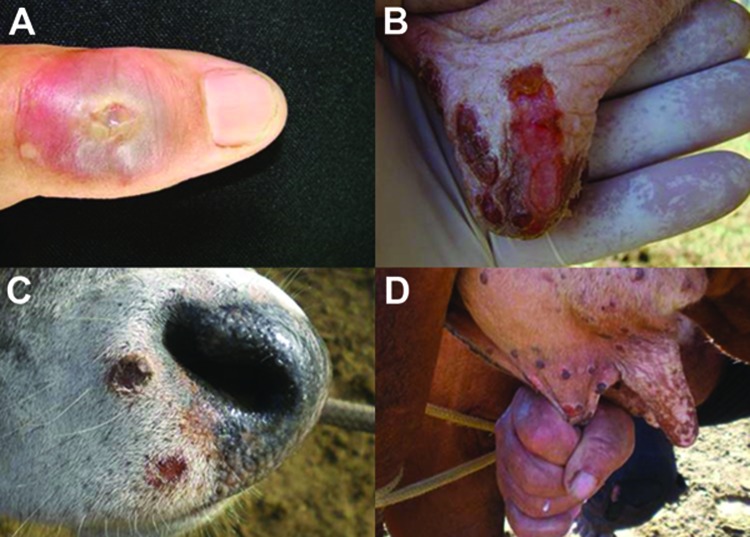
Exanthematic lesions caused by vaccinia virus (VACV) infection during this outbreak. A) Vesicular lesion on milker’s finger that advanced to an ulcerative stage. B and D) Typical lesions on teats and udder of a dairy cow infected by VACV at different stages, ranging from ulceration to scabs. C) Lesions on a calf’s muzzle probably caused by VACV infection during suckling.

Dairy cattle and workers were affected. However, the source (index case) of this outbreak was not identified. At the study site, 44 lactating dairy cows became sick and had painful vesicular lesions on teats, udders, and inner thighs that rapidly progressed to ulcerative lesions and scabs (Figure 2, panels B and D). Two animals had extensive necrosis because of secondary infections, which led to loss of teats. Lesions were also observed on lips, muzzles, oral cavities, and tongues of calves (Figure 2, panel D). Three dairy workers became sick during the outbreak after direct contact with sick animals and had typical orthopoxvirus (OPV) lesions on their hands, forearms, and abdomen (Figure 2, panel A). Pain in the lesion region, fever, and fatigue were also reported by sick patients.

We collected 4 scabs and 44 serum samples from the 44 sick animals and 3 serum samples from the 3 dairy workers. Serum samples were tested by using 50% plaque reduction neutralization tests as described (*8*). Neutralizing antibodies were detected in 40 (90.0%) bovine and in 3 (100%) human samples, and titers ranged from 20 to 640 neutralizing units/mL. Scabs were macerated in buffer and centrifuged. Supernatants were diluted 1:100 in phosphate-buffered saline and used in a nested PCR specific for the C11R viral growth factor gene as described (*9**,**10*). OPV-specific fragments from 2 scab samples were amplified. Samples were also subjected to virus isolation in Vero cells.

We isolated virus from 1 of the samples that was positive for viral growth factor by nested PCR. Negative results for VACV by PCR and virus isolation might have been caused by loss of virus titers and DNA degradation during sample transportation. After a typical poxvirus cytopathic effect was observed, virus was plaque purified and placed on Vero cell monolayers for viral amplification. This new VACV isolate was named Pará virus (PARV).

To investigate the relationship between PARV and BAV, virus gene A56R (hemagglutinin) was amplified and sequenced (*11*). The A56R gene is traditionally used for phylogenetic analysis. In addition, PARV A26L (A-type inclusion body) was also sequenced (*12*). The PCR fragments obtained were directly sequenced in both orientations and in triplicate by using a Mega-BACE 1000 Sequencer) (GE Healthcare, Little Chalfont, UK). Sequences were aligned with OPV sequences from GenBank by using ClustalW (www.ncbi.nlm.nih.gov/pmc/articles/PMC308517), and alignments were manually checked with MEGA version 4.0 software (Arizona State University, Phoenix, AZ, USA).

**Figure 3:**
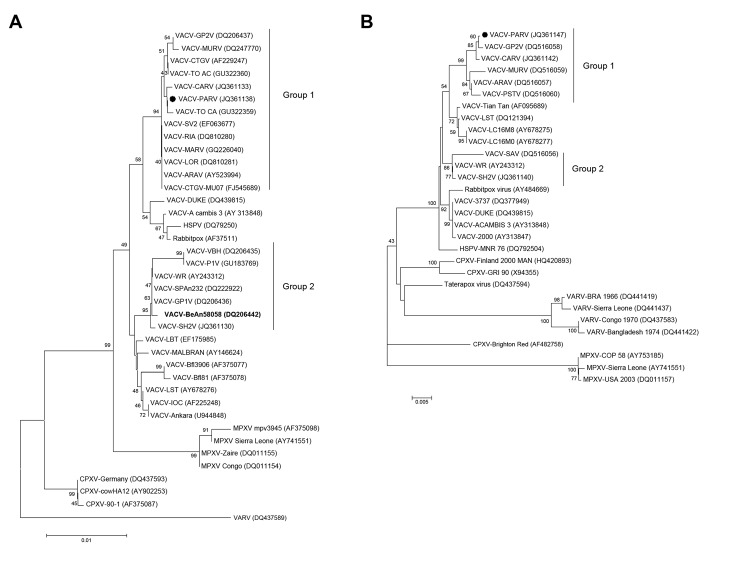
Phylogenetic trees based on orthopoxvirus nucleotide sequences of A56R (A) and A26L (B) genes of vaccinia virus (VACV), Pará State, Brazil. Pará virus (PARV) clusters with VACV group 1 from Brazil. Phylogenetic analysis showed that PARV (black dots) clustered in the VACV-BR-G1 clade and that BeAN58058 virus (BAV) clustered in the VACV-BR-G2 clade. A26L sequence was obtained only from PARV. Trees were constructed by using the neighbor-joining method, the Tamura-Nei model of nucleotide substitutions, and bootstrap of 1,000 replicates in MEGA version 4.0 software (Arizona State University, Phoenix, AZ, USA). In panel A, BAV is shown in **boldface**. GenBank accession numbers are indicated in parentheses. Values along the branches indicate bootstrap values. Scale bars indicate nucleotide substitutions per site. GP2V, Guarani P2 virus; MURV, Muriae virus; CTGV, Cantagalo virus; CARV, Carangola virus; MARV, Mariana virus; ARAV, Araçatuba virus; HSPV, horsepox virus; GP1V, Guarani P1 virus; MPXV, monkeypox virus; PSTV, Passatempo; CPXV, cowpox virus; VARV, variola virus.

Optimal alignment of the A56R gene showed high identity among PARV and several group 1 VACV-BR isolates (average identity 99.8%), including VACV-TO CA (GU322359) (identity 99.9%), an amplicon obtained from blood of an Amazon monkey in Tocantins State, Brazil, in 2002 (Figure 3). PARV and BAV showed 98.3% identity with each other. PARV also showed a signature deletion of 18 nt that was also present in A56R sequences of other group 1 VACV-BR isolates 

Phylogenetic trees of the A56R (Figure 3, panel A) or A26L (Figure 3, panel B) genes were constructed by using the neighbor-joining method, 1,000 bootstrap replicates, and the Tamura 3-parameter model (MEGA version 4). PARV sequences clustered with several group 1 VACV-BRs isolated during several bovine vaccinia outbreaks in Brazil. In both trees, PARV clustered in group 1 VACV-BR, whereas BAV clustered in group 2.

## Conclusions

Our results confirm circulation of a new group 1 VACV-BR isolate in Pará State in the Amazon region of Brazil in the same location where BAV (group 2 VACV-BR) was isolated (*6**,**7*). In recent years, Bovine vaccinia outbreaks in southeastern Brazil rapidly spread to neighboring states (*1*). Epidemiologic studies suggest that movement of sick humans and the animal trade are the main causes of this circulation (*1*). The relevance of VACV circulation in the context of bovine vaccinia outbreaks has been discussed (*13*).

Several isolates belong to group 1, which is most commonly isolated from sick cattle or cow milkers; some isolates were detected in peridomestic rodents and wild monkeys (*8**,**13*). Therefore, although our data demonstrated that PARV does not cluster with BAV, it is not possible to phylogenetically define which group 1 isolate specifically generated PARV or caused the outbreak because of limited number of available gene sequences from VACV-BR isolates. Nevertheless, we believe the presence of this new isolate in Pará State likely resulted from virus spread from Tocantins, Maranhão, or Mato Grosso, 3 neighboring states of Pará State, which had Bovine vaccinia outbreaks in recent years, and not from reemergence of BAV (*1*).

Although group 2 VACV-BR isolates, including BAV, have not been detected in the Amazon region of Brazil in recent years, we believe that these viruses may be silently circulating or associated with bovine vaccinia outbreaks. As in other regions in Brazil, VACV outbreaks are underreported in the Amazon region because of its large size and the natural complexity involved in surveillance of zoonotic diseases. Our results reinforce the need for studies on VACV diversity and its transmission chain, which would be useful for the Amazon region in Brazil.
